# Re-irradiation of adrenal metastases using MR-guided adaptive SABR: A feasible and effective approach in a high-risk population^☆^

**DOI:** 10.1016/j.ctro.2025.101056

**Published:** 2025-10-12

**Authors:** Miguel A. Palacios, Anna M.E. Bruynzeel, Peter S.N. van Rossum, Omar Bohoudi, Suresh Senan, Famke L. Schneiders

**Affiliations:** a Department of Radiation Oncology, Amsterdam UMC, Amsterdam, The Netherlands; b Cancer Treatment and Quality of Life, Cancer Center Amsterdam, Amsterdam, The Netherlands

**Keywords:** Re-irradiation, Adrenal metastasis, SABR, MR linac, Oligometastasis

## Abstract

Re-irradiation of adrenal metastases is challenging due to cumulative dose constraints. We present seven patients treated with MR-guided adaptive stereotactic ablative radiotherapy (SABR), leveraging real-time imaging and on-table adaptation for precise dose delivery (5× 10 Gy or 3× 15 Gy). This first report of ablative-dose MR-guided SABR in re-irradiation demonstrates excellent local control and acceptable toxicity, with six patients progression-free at median 8.6 months and no grade ≥3 events (one grade 2 vertebral fracture, one grade 1 nausea). By providing a non-invasive alternative to surgery or palliative radiotherapy, MR-guided adaptive SABR expands therapeutic options for high-risk patients.

## Highlights

• Local recurrence after SABR for adrenal metastasis is challenging.
• First report of ablative-dose re-SABR (5 × 10  Gy or 3 × 15  Gy) in this setting.
• Six of seven patients were progression-free at median 8.6 months follow-up.
• No grade ≥ 3 toxicities; only one grade 2 vertebral fracture and one grade 1 nausea.
• MR-guided adaptive re-SABR provides a tolerable, non-invasive, high-precision treatment option.

## Introduction

Oligometastatic disease is generally characterized by the presence of one to five metastatic lesions (with the primary tumor status being optionally counted as lesion), provided that every metastatic site can be treated safely [1]. The adrenal glands are common sites for metastatic disease [2] and stereotactic ablative radiotherapy (SABR) is an established modality for treating oligometastatic disease, including adrenal metastases, providing effective local control with minimal morbidity [[3], [4], [5]]. However, the treatment of lesions which recur after SABR can pose a challenge. Re-irradiation of adrenal metastases is particularly challenging due to cumulative radiation dose constraints for surrounding organs-at-risk (OARs).

Traditionally, options for managing recurrent adrenal metastases include surgical resection, palliative radiotherapy or systemic therapy. Surgical salvage for recurrences after SABR can be more difficult due to radiation-induced changes in tissue planes and vascularity. The present study assesses the feasibility and safety of re-irradiation of adrenal metastases using ablative MR-guided SABR at a single institution, using real-time imaging and adaptive radiotherapy planning to enhance treatment precision [6].

## Materials and methods

### Study population

Using an institutional Ethics-approved database we identified seven consecutive patients who were treated using MR-guided SABR re-irradiation between March 2024 and March 2025. All patients had local recurrence or oligoprogressive disease following prior SABR or high-dose hypofractionated radiotherapy to the adrenal gland and were eligible for SABR according to multidisciplinary review. Patient demographics, primary tumor site, time since prior SABR, and concurrent treatments, were extracted from electronic medical records.

### Treatment

Re-irradiation was delivered with the MRIdian MR-Linac system (ViewRay Inc.), using daily online MRI-based adaptive planning, breath-hold gating, and real-time imaging to ensure precise dose delivery and OAR sparing [6]. Daily MRI scans enabled target recontouring and plan adaptation based on anatomical changes. Gross tumor volume (GTV) was defined as the entire adrenal gland including the metastasis. A planning target volume (PTV) margin of 3 mm allowed for dose escalation while respecting OAR constraints. Plans were reviewed and approved in an online adaptive workflow involving radiation oncologists, physicists, and radiation therapists. A fasting period of 2–3 h was instructed prior to each treatment fraction.

Re-irradiation was delivered in five fractions of 10  Gy (biologically effective dose, BED_10_, of 100  Gy) in five patients. Another patient received three fractions of 15  Gy (BED_10_ = 112.5  Gy), and one patient completed only three fractions of 10  Gy (BED_10_ = 60  Gy) due to declining clinical condition. The biologically effective dose (BED) and equivalent dose in 2-Gy fractions (EQD2) were calculated using an α/β ratio of 10  Gy for tumor tissue and 2–3  Gy for late-responding normal tissues, depending on the organ. Dose constraints for critical OARs—including the stomach, duodenum, bowel, kidney, and spinal cord—were applied according to the AAPM TG-101 SBRT guidelines [7]. Retrospective dosimetric comparisons were conducted between adaptive and non-adaptive (reference) plans.

### Cumulative dose methodology and uncertainties

To estimate cumulative organ-at-risk (OAR) dose from initial and re-irradiation treatments, we performed a dose accumulation workflow tailored to the imaging modality of the first course. For patients initially treated on an MR-linac, deformable image registration (DIR) of the first dose distribution onto the second planning MR was performed, minimizing inter-modality uncertainty. For patients treated on a CT-based linac, DIR to the second planning MR introduced greater uncertainty due to differences in image contrast.

All registrations were performed in Velocity AI v4.1. DIR quality was assessed by visual inspection of deformation vector fields and, where feasible, Dice Similarity Coefficient (DSC) calculations for relevant OARs. To increase robustness, at least three different DIR algorithms were compared for each patient, especially when anatomical changes or low DSC values were observed. Final cumulative dose estimates for abdominal OARs reflected consensus review by both physician and physicist.

Importantly, cumulative dose estimates were used for retrospective analysis only, not for prescription. For treatment planning, a conservative worst-case approach was adopted, assuming geometric overlap of maximum dose points (e.g., D0.03 cc) from both treatments. Accordingly, re-irradiation prescription doses were reduced to ensure combined OAR exposure remained within established safety limits.

### Outcome measures

Follow-up CT imaging was performed at approximately 3, 6, and 12 months post-treatment. Radiologic response was assessed by RECIST 1.1 criteria. Adverse events were scored using the Common Terminology Criteria for Adverse Events (CTCAE) version 5.0. Local failure was defined as progression within the treated adrenal volume. Progression-free survival (PFS) and overall survival (OS) were calculated from the end of re-irradiation. Given the small sample size and limited number of events, time-to-event medians were expected to be non-estimable by Kaplan–Meier (KM). Instead, we therefore prioritized descriptive reporting using per-patient timelines in a swimmer plot.

## Results

The cohort comprised seven patients with a median age of 64 years (range: 50–75) and WHO performance status ranged from 0 to 2. All but one patient had a primary lung cancer, with one having a primary renal cell carcinoma [Table 1]. Prior ablative radiotherapy schemes (5 out of 7 patients received treatment in other institutes) included a single dose of 15 or 24  Gy, a fractionated schedule (e.g., 5 × 7 Gy, 5 × 10  Gy, 5 x 9.4 Gy), and in one case, three prior treatments had targeted the left adrenal gland and adjacent regions. The interval between initial and new SABR ranged from 13.8 to 49.0 months, with a median of 21.6 months. Two patients undergoing systemic therapies with pemetrexed and osimertinib, respectively, temporarily discontinued these during SABR reirradiation. According to the ESTRO/EORTC consensus on re-irradiation reporting, all cases were classified as re-irradiation type 1, indicating geometrical overlap between the new and previously irradiated treatment volumes [8].

**Table 1:** Patient characteristics, including prescribed dose of first course, second (re-irradiation course) and dose constraints for stomach, bowel and duodenum used during re-irradiation. NSCLC = non-small cell lung cancer, RT = radiotherapy, SCLC = small cell lung cancer. Cells with a line (–) indicate that the cumulative dose to the specified organ was not calculated. This was the case for organs with high inter-fractional mobility or those located at a sufficient distance from the target, as the dose received was considered to be clinically insignificant and well below established tolerance limits.

Patient	1	2	3	4	5	6	7
Primary	SCLC	NSCLC	NSCLC	NSCLC	NSCLC	SCLC	Renal
Side	bilateral	left	right	right	left	left	left
Previous RT	5x8Gy	5x7Gy	1x24Gy	5x10Gy	1 x 15 Gy + 4 x 10 Gy	5x7Gy	5x10Gy
Re-irradiation RT	5x10Gy	5x10Gy	3x15Gy	3x10Gy	5x10Gy	5x10Gy	5x10Gy
Constraints used for RT2 (re-irradiation)							
Stomach (0.1 cc)	22.5 Gy	36.0 Gy	27.0 Gy	27.0 Gy	15.0 Gy	27.5 Gy	30.0 Gy
Bowel (0.1 cc)	22.5 Gy	22.5 Gy	27.0 Gy	27.0 Gy	15.0 Gy	35.0 Gy	36.0 Gy
Duodenum (0.1 cc)	22.5 Gy	22.5 Gy	27.0 Gy	27.0 Gy	15.0 Gy	10.0 Gy	36.0 Gy
Accumulated EQD2 dose for OARs (Gy)							
Stomach (0.1 cc)	76.75	34.12	–	67.09	61.54	60.62	71.21
Bowel (0.1 cc)	75.56	71.26	–	–	55.38	–	–
Duodenum (0.1 cc)	–	–	37.14	–	–	70.15	–

Cells with a line (–) indicate that the cumulative dose to the specified organ was not calculated. This was the case for organs with high inter-fractional mobility or those located at a sufficient distance from the target, as the dose received was considered to be clinically insignificant and well below established tolerance limits.

The median follow-up after reirradiation was 8.6 months (range: 1.2–14.4 months). At the time of analysis all patients were alive, with a single patient who developed diffuse disease progression at 8.6 months and subsequently started with next-line systemic treatment. The other six patients remained progression-free both locally and outside the adrenal gland at last follow-up. Given one observed event and short follow-up, KM medians were non-estimable; detailed per-patient timelines are shown in Fig. 2.

CT imaging at three months post-SABR was obtained in six patients; despite minor variations in lesion size, all lesions fulfilled RECIST 1.1 criteria for stable disease. At six months, follow-up CT was available for four patients and again demonstrated stable disease in all cases. Twelve-month imaging was performed in three patients, each of whom continued to exhibit stable disease. One patient was transferred to hospice care and therefore did not undergo further imaging.

In a patient who developed progressive pain beginning three weeks after treatment, a vertebral fracture adjacent to the treated volume was identified 7.8 months post-SABR, classified as CTCAE Grade 2. The shortest distance from the target to the vertebra in this patient was 0.5 cm, and the estimated delivered cumulative EQD2 to 1.0 cc of the vertebra after all treatment courses was 101 Gy. This patient was managed conservatively with pain blockade by the pain team. Another patient experienced a single episode of vomiting following treatment, classified as CTCAE Grade 1. No further episodes of vomiting occurred after prophylactic anti-emetics were administered. A detailed toxicity timeline, including onset, management, and follow-up, is provided in Supplementary Table S1.

### Dosimetric analysis

**Fig. 1:**
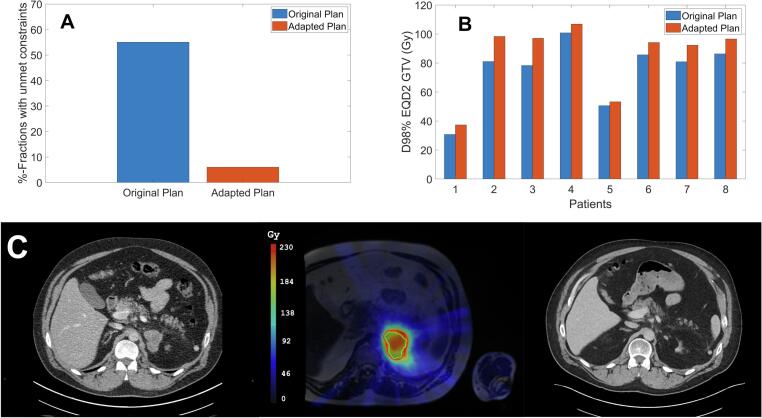
Percentage of fractions exceeding organ-at-risk (OAR) dose constraints for the original treatment plan compared to the online adaptive plan (A). Cumulative EQD2 (Gy) to 98% of the gross tumor volume (GTV) for each patient, comparing the original treatment plan with the online adaptive plan (B). Contrast-enhanced CT imaging prior to the initial treatment course in 2021, revealing a metastasis in the left adrenal gland (C-left); total EQD2 (α/β10 for tumor and α/β3 for OARs) delivered dose distribution following three stereotactic body radiotherapy (SBRT) courses (C-middle); and post-treatment contrast-enhanced CT imaging in 2025 (C-right).

The median GTV at the time of re-irradiation simulation was 9.9 cc (range: 5.7 – 23.9 cc). A median GTV increase of 1.9 cc from the time of simulation was observed at the first SABR fraction. The median time that elapsed between simulation and the first SABR fraction was 26 days (range: 17 – 31 days). Intra-treatment MR-imaging revealed that 6 lesions exhibited radiological regression, whereas a lesion treated with only 3 fractions of 10 Gy showed a slight increase in size at the end of treatment. During treatment, 39 % of all fractions required an online correction of patient position to account for intra-fractional anatomical changes. The median applied online 3D vector correction amounted 0.5 cm (range: 0.2 – 3.6 cm). In 55 % of all fractions, the original plan failed to meet the OAR constraints, and this was only seen for left-sided adrenal tumors. After online plan adaptation, only in 6 % of the fractions the new plan did not meet the OAR constraints (and only to a very small degree). However, this did not lead to a cumulative excess of the OAR dose limits over the whole treatment course. Adapted plans also led to improved PTV coverage. Fig. 1B shows that the cumulative D98% in EQD2 of GTV improved for all patients in the adapted plans with respect to the original plan. Similarly, the V95% of PTV showed a median increase of 6.2 % (range: 0.4–28.3 %) in coverage for all patients.

**Fig. 2:**
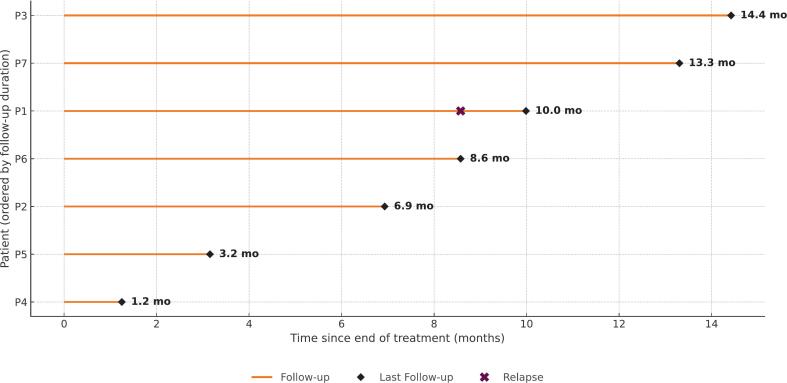
Swimmer’s plot illustrating follow-up duration for all patients (n = 7). Timepoint 0 corresponds to the end of treatment. Orange lines represent follow-up until last contact (black diamond). A distant relapse is indicated for Patient 1 (purple cross). Follow-up duration in months is shown at the end of each line, with patients ordered by follow-up length. (For interpretation of the references to colour in this figure legend, the reader is referred to the web version of this article.)

To ensure stringent OAR constraints were respected in the re-irradiation setting, a compromise in PTV coverage was necessary in 30 % of the fractions delivered to left adrenal gland patients. As shown in Fig. 1, dose compromise was most notable in Patients 1 and 5, who had tumors adjacent to critical OARs. These clinical decisions were made by assigning a higher priority to the safety of adjacent OARs over target coverage. The compromise was also reflected in the reduction of the near-minimum dose to the GTV, specifically the D98%, to ensure OAR dose limits were met.

Fig. 1C shows an example of a contrast-enhanced diagnostic CT of a patient with a left-sided adrenal metastasis before initial SABR and after re-re-irradiation. This patient had to interrupt the initial treatment course after receiving a first fraction of 15 Gy due to adrenal hemorrhage. Subsequent local tumor progression 7 months later was treated with a second course of SABR to a dose of 40 Gy in 4 fractions. Forty months later, the patient developed another in-field recurrence. The cumulative EQD2 (Gy) after a third course of 5x10Gy is shown in Fig. 1.

## Discussion

Isolated in-field recurrences after SABR for oligometastatic disease is not an uncommon clinical presentation. A previous *meta*-analysis reported 1- and 2-year local failure rates after SABR of 18 % and 37 %, respectively [5]. There is limited published data available on use of SABR for adrenal relapses, and even primary SABR for adrenal metastases can be challenging due to the proximity of critical organs. Recent advances in MR-guided techniques have demonstrated that it is feasible to safely deliver high-dose SABR—with the option to intentionally underdose small volumes adjacent to critical structures—while achieving excellent local control [4]. The main findings of the present study are that MR-guided SABR enabled the safe delivery of SABR in the re-irradiation setting. Online plan adaptation reduced the doses to the OARs, while improving target coverage at the same time, especially for left-sided adrenal tumors. While physician-reported toxicity was carefully documented with a detailed timeline of onset, management, and follow-up, patient-reported outcomes were not collected, which represents a limitation of the study.

Only limited data on adrenal re-irradiation have been published to date. Mills et al. [9] reported three re-irradiation cases within a multi-institutional MR-guided adrenal SABR cohort, treated with either 60 Gy in 10 fractions (BED_10_ ≈ 96 Gy) or 40 Gy in 5 fractions (BED_10_ ≈ 72 Gy), without grade ≥ 3 toxicity. Chawla et al. [10] described one patient undergoing a second course of adrenal SBRT after in-field recurrence, although the exact dose was not specified; in their overall series, doses ranged from 16–50 Gy in 4–16 fractions (median 40 Gy), with no grade ≥ 3 toxicity observed. Most of our patients were treated with 5 × 10 Gy (BED_10_ = 100 Gy). This BED_10_ is higher than in previously published series yet appeared to be delivered safely with MR-guided adaptive SABR, again without grade ≥ 3 toxicity.

Non-adaptive re-irradiation techniques are often either not feasible due to anatomical complexity and the associated risk of severe toxicity, or not effective because the prescribed dose must be lowered to avoid potential toxicity. Here we demonstrate the potential of MR-guided SABR for reirradiation, particularly in anatomically complex regions such as the abdomen. MR-guided SABR provides superior soft-tissue contrast and real-time imaging, enabling daily adaptive planning and precise dose delivery, which are particularly important when treating previously irradiated areas where the therapeutic window is narrow. This is especially relevant for adrenal metastases, where continuous motion and deformation of surrounding OARs complicate safe reirradiation. Additionally, the integration of high-quality imaging before and during treatment allows for accurate reconstruction of previously delivered dose distributions. This facilitates better estimation of cumulative organ tolerances and supports individualized plan adaptation, enhancing both safety and efficacy in the reirradiation setting. In this setting it is necessary to evaluate each case independently and prescribe individualized dose constraints. Each patient in this study was planned with individualized OARs constraints, taking previously irradiated courses into account. Caution remains critical regarding potential vertebral fractures, underscoring the importance of adhering strictly to vertebral dose constraints and avoiding overlap with prior radiation fields.

This short communication describes a safe and feasible MR-guided SABR approach for re-irradiation of adrenal metastases. With precise targeting of lesions and plan adaptation to account for anatomical variations, our preliminary results indicate favorable local control rates with acceptable toxicity, thus potentially expanding therapeutic options for patients previously treated with radiotherapy. Results should be interpreted as preliminary given the limited cohort size, short follow-up, and lack of a comparator. Larger multi-institutional studies are required to validate these early findings.

## Conclusion

MR-guided adaptive SABR is a feasible and effective re-irradiation strategy for adrenal metastases, providing high local control and low toxicity through real-time imaging and adaptive planning. This approach expands non-invasive treatment options for challenging re-irradiation scenarios.

## Declaration of Competing Interest

The authors declare that they have no known competing financial interests or personal relationships that could have appeared to influence the work reported in this paper.

## References

[1] Lievens Y., Guckenberger M., Gomez D., Hoyer M., Iyengar P., Kindts I. (2020 Jul). Defining oligometastatic disease from a radiation oncology perspective: An ESTRO-ASTRO consensus document. Radiother Oncol..

[2] Spartalis E., Drikos I., Ioannidis A., Chrysikos D., Athanasiadis D.I., Spartalis M. (2019). Metastatic carcinomas of the adrenal glands: From diagnosis to treatment. Anticancer Res.

[3] Ugurluer G., Schneiders F.L., Corradini S., Boldrini L., Kotecha R., Kelly P. (2024). Factors influencing local control after MR-guided stereotactic body radiotherapy (MRgSBRT) for adrenal metastases. Clin Transl Radiat Oncol.

[4] Schneiders F.L., van Vliet C., Giraud N., Bruynzeel A.M.E., Slotman B.J., Palacios M.A. (2023). Clinical outcomes of MR-guided adrenal stereotactic ablative radiotherapy with preferential sparing of organs at risk. Clin Transl Radiat Oncol.

[5] Chen W.C., Baal J.D., Baal U., Pai J., Gottschalk A., Boreta L. (2020). Stereotactic Body Radiation Therapy of Adrenal Metastases: A Pooled Meta-Analysis and Systematic Review of 39 Studies with 1006 Patients. Int J Radiat Oncol Biol Phys.

[6] Palacios M.A., Bohoudi O., Bruynzeel A.M.E., van Sörnsen-de Koste J.R., Cobussen P., Slotman B.J. (2018). Role of daily plan adaptation in MR-guided stereotactic ablative radiotherapy for adrenal metastases. Int J Radiat Oncol.

[7] Benedict SH, Yenice KM, Followill D, Galvin JM, Hinson W, Kavanagh B, Keall P, Lovelock M, Meeks S, Papiez L, Purdie T, Sadagopan R, Schell MC, Salter B, Schlesinger DJ, Shiu AS, Solberg T, Song DY, Stieber V, Timmerman R, Tomé WA, Verellen D, Wang L, Yin FF. Stereotactic body radiation therapy: the report of AAPM Task Group 101. Med Phys. 2010 Aug;37(8):4078-101. doi: 10.1118/1.3438081. Erratum in: Med Phys. 2012 Jan;39(1):563. Dosage error in article text. Erratum in: Med Phys. 2023 Jun;50(6):3885. doi: 10.1002/mp.16159. PMID: 20879569. 10.1118/1.3438081 20879569

[8] Andratschke N, Willmann J, Appelt AL, Alyamani N, Balermpas P, Baumert BG, Hurkmans C, Høyer M, Langendijk JA, Kaidar-Person O, van der Linden Y, Meattini I, Niyazi M, Reynaert N, De Ruysscher D, Tanadini-Lang S, Hoskin P, Poortmans P, Nieder C. European Society for Radiotherapy and Oncology and European Organisation for Research and Treatment of Cancer consensus on re-irradiation: definition, reporting, and clinical decision making. Lancet Oncol. 2022 Oct;23(10):e469-e478. doi: 10.1016/S1470-2045(22)00447-8. Erratum in: Lancet Oncol. 2022 Nov;23(11):e492. doi: 10.1016/S1470-2045(22)00609-X. PMID: 36174633. 10.1016/S1470-2045(22)00447-8 36174633

[9] Mills M., Kotecha R., Herrera R., Kutuk T., Fahey M., Wuthrick E. (2024 Jan). Multi-institutional experience of MR-guided stereotactic body radiation therapy for adrenal gland metastases. Clin Transl Radiat Oncol..

[10] Chawla S., Chen Y., Katz A.W., Muhs A.G., Philip A., Okunieff P. (2009 Sep 1). Stereotactic body radiotherapy for treatment of adrenal metastases. Int J Radiat Oncol Biol Phys..

