# Supplementary material for: Re-irradiation of adrenal metastases using MR-guided adaptive SABR: A feasible and effective approach in a high-risk population
Source: Clin Transl Radiat Oncol. 2025 Oct 12;56:101056. doi: 10.1016/j.ctro.2025.101056 (PMC12552146; doi:10.1016/j.ctro.2025.101056)
Supplement: Supplementary Data 1 [file mmc1.docx]

# Supplementary Table S1 - Toxicity Timeline

| **Patient ID** | **Toxicity** | **CTCAE Grade** | **Onset (time after SABR)** | **Management** | **Outcome** |
| --- | --- | --- | --- | --- | --- |
| 2 | Vomiting | 1 | Day of treatment | Prophylactic anti-emetics | No recurrence |
| 3 | Vertebral fracture | 2 | 3 weeks (pain), diagnosed 7.8 months | Pain team blockade, conservative | Ongoing pain control, stable disease |
